# DNA Damage: From Chronic Inflammation to Age-Related Deterioration

**DOI:** 10.3389/fgene.2016.00187

**Published:** 2016-10-25

**Authors:** Anna Ioannidou, Evi Goulielmaki, George A. Garinis

**Affiliations:** ^1^Institute of Molecular Biology and Biotechnology, Foundation for Research and Technology-HellasHeraklion, Greece; ^2^Department of Biology, University of CreteHeraklion, Greece

**Keywords:** DNA damage, DNA repair, inflammation, aging, cancer

## Abstract

To lessen the “wear and tear” of existence, cells have evolved mechanisms that continuously sense DNA lesions, repair DNA damage and restore the compromised genome back to its native form. Besides genome maintenance pathways, multicellular organisms may also employ adaptive and innate immune mechanisms to guard themselves against bacteria or viruses. Recent evidence points to reciprocal interactions between DNA repair, DNA damage responses and aspects of immunity; both self-maintenance and defense responses share a battery of common players and signaling pathways aimed at safeguarding our bodily functions over time. In the short-term, this functional interplay would allow injured cells to restore damaged DNA templates or communicate their compromised state to the microenvironment. In the long-term, however, it may result in the (premature) onset of age-related degeneration, including cancer. Here, we discuss the beneficial and unrewarding outcomes of DNA damage-driven inflammation in the context of tissue-specific pathology and disease progression.

## Maintenance and defense: twice the deal

To withstand the hazards of existence, multicellular organisms need to preserve their bodily functions for long periods of time and protect themselves against pathogens. Taking the cell as a point of reference, the maintenance is directed inwards to counteract macromolecular damage. This often involves restoring injured nucleic acids back to their native form (Hoeijmakers, 2001) or replenishing proteins and lipids once damaged by harmful byproducts of metabolism (Balaban et al., 2005). Instead, cellular defense mechanisms, such as the innate immune responses are mainly directed outwards to protect the organism against irritants, pathogens, or injured cells.

**Figure 1:**
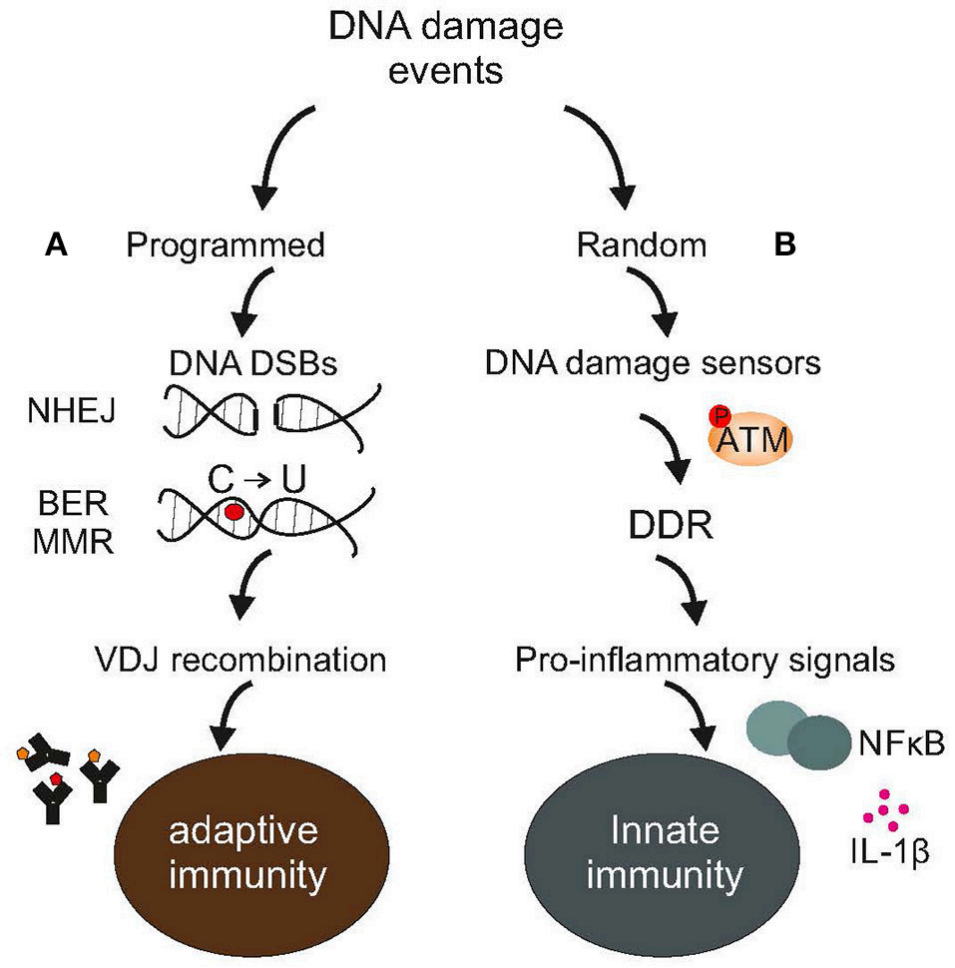
**DNA damage events and the immune system**. **(A)** Adaptive immunity relies on programmed DNA damage events and error-prone DNA repair mechanisms, such as the NHEJ to increase antibody diversity during VDJ recombination in developing lymphocytes or the BER or MMR to turn cytosines into uracils in Ig genes **(B)**. Random DNA damage events trigger the activation of DDR-driven pro-inflammatory signals, including NFkB or various interleukins leading to chronic inflammation and disease. The figure is a summarization/scheme of the manuscript.

Since the problem of damage or the invasion of cells by pathogens has existed nearly *ab initio*, maintenance and defense must have arisen early during evolution. Indeed, even simple unicellular organisms such as bacteria possess multiple caretaking systems or enzymes that protect against viral infections and pathogens (Žgur-Bertok, 2013); remarkably, some prokaryotes employ a structurally distinct family of nucleases with a dual function e.g., in DNA repair and antiviral immunity (Babu et al., 2011). Similar to bacteria, mammals provide ample evidence that mechanisms of DNA repair and immunity have evolved together (Alt et al., 2013). For example, non-homologous end-joining is involved in the development of lymphocytes in resolving recombination intermediates i.e., DNA strand breaks (DSBs) that occur during V(D)J recombination (Boboila et al., 2012). Likewise, “programmed” DNA lesions followed by error-prone DNA repair dramatically increase antibody diversity by triggering somatic hypermutation of immunoglobulin variable genes (Di Noia and Neuberger, 2007). Activation-induced cytidine deaminase is a unique enzyme that deaminates cytosines into uracils in Ig genes. Direct replication over uracils may lead to C → T transition mutations. Moreover, removal of the uracils by base-excision repair (BER) (Krokan and Bjoras, 2013) generates abasic sites; replication bypass of abasic sites may also lead to mutations (Di Noia and Neuberger, 2007). The relevance of BER enzymes in antibody gene diversification is revealed in patients carrying a defect in uracil N glycosylase that show marked deficiencies in immunoglobulin (Ig) class-switch recombination and somatic hypermutation generation (Imai et al., 2003). Alternatively, mismatch repair (MMR) (Pena-Diaz and Jiricny, 2012) could recruit polη for error-prone repair of U/G mismatches further promoting mutations in immunoglobulin variable genes (Di Noia and Neuberger, 2007). Nonetheless, the evolutionary transition from one-celled microbes to more complex living systems has pushed for drastic changes in maintenance and defense strategies. In mammals, a single fertilized egg rapidly divides into several trillions of cells grouped into specialized tissues with marked differences in terms of developmental origin, regenerative capacity and ability to cope with damage. Moreover, tissues, organs and organ systems team up to perform specific tasks such as the body's first line of defense against bacteria or viruses. This inherent complexity arising from manifold levels of organization within multicellular life forms requires that genome maintenance, the DNA damage response (DDR) and defense strategies are tightly linked (Velimezi et al., 2013) and highly coordinated processes (Figure 1).

## Linking DDR with pro-inflammatory nuclear factors

Unlike adaptive immunity, DNA repair mechanisms *per se* do not seem to play a role in innate immunity (Xu, 2006). However, innate immune cells e.g., natural killer (NK) cells, natural killer T (NKT) cells, γδ T cells or phagocytes often rely on DDR to activate nuclear factors (Liu et al., 1996; Frontini et al., 2009), cell surface ligands (González et al., 2008), intercellular adhesion molecules (Gorgoulis et al., 2003), or smaller peptides i.e., cytokines or chemokines in response to stress (Kuilman and Peeper, 2009). A major step forward linking DDR with pro-inflammatory nuclear factors was the discovery that DNA damage activates cytoplasmic NF-κB (nuclear factor kappa-light-chain-enhancer of activated B cells) (Hayden and Ghosh, 2008); NF-κB is a fast-acting transcription factor present in most cells as a dimer of RelA or p65, c-Rel, RelB, p50, and p52 subunits (Hayden and Ghosh, 2008). In case of DNA damage, the “nuclear-to-cytoplasmic” response originates mainly from DNA double strand breaks (DSBs) that trigger the SUMO (small ubiquitin like modifier) modification of NEMO (NF-κB essential modulator) in the nucleus (Huang et al., 2003). In turn, the DNA damage sensor ATM (Ataxia telangiectasia, mutated) kinase phosphorylates SUMOylated NEMO triggering the removal of SUMO and the addition of a ubiquitin residue. These events allow the export of ATM-NEMO complex out of the nucleus to activate NF-κB in the cytoplasm through the stimulation of the canonical inhibitor of κB (IκB) kinase (IKK) complex and IκB degradation (Scheidereit, 2006; Wu et al., 2006). Disruption of the sumoylation sites on NEMO abolishes the activation of IKK complex upon DNA damage. Following the degradation of IκB, the NF-κB (p65/p50) heterodimer enters the nucleus and modulates the expression of target genes (Karin, 2006). Other stress stimuli, such as oxidative stress or heat shock may also induce the SUMOylation of NEMO in an ATM-independent manner (Li et al., 2001; Oeckinghaus et al., 2011) suggesting that NF-κB activation is a conserved survival response that is not restricted to DDR. Intriguingly, NF-κB play roles in DNA repair itself; for instance, *p65*^−/−^ mouse embryonic fibroblasts show aberrant chromosomal structures that resemble those seen in Fanconi anemia patients (D'Andrea and Grompe, 2003) or the Bloom syndrome (Furuichi, 2001). Moreover, NF-κB activates the CtIP–BRCA1 complex to trigger DNA-end processing (Volcic et al., 2012). Lastly, DDR-mediated activation of ATM-NEMO-NF-κB pathway plays a physiological role during lymphocyte development in response to “programmed” DSBs (Bredemeyer et al., 2008). Similar to NF-κB, the interferon regulatory factors (IRFs) make up another family of immune-related transcription factors with a role in DNA repair. IRF-1 appears to regulate the DNA inter-strand cross-link (ICL) repair pathway (Frontini et al., 2009). Moreover, IRF-3 is an *in vivo* target of DNA-PK (Karpova et al., 2002), a protein with well-established functions in the DNA repair and V(D)J recombination (Lieber et al., 2003). Finally, IRF-5 is a direct transcriptional target of p53 also upon exposure to various genotoxic agents (Mori et al., 2002). IRFs maintain functionally diverse roles in interferon-induced antiviral defense (e.g., IRF-1, IRF-3, and IRF-7) (Akira et al., 2006; Paun and Pitha, 2007), lymphocyte development (e.g., IRF-4) (Lu et al., 2003), macrophage-induced inflammation (e.g., IRF-8) (Paun and Pitha, 2007), or keratinocyte differentiation (e.g., IRF-6) (Richardson et al., 2006). IRFs induce the transcription of type I interferons and other pro-inflammatory cytokines by recognizing a consensus IFN-stimulated response element on the promoter of target genes (Taniguchi et al., 2001). IRF-1 is required for oncogene-induced apoptosis of embryonic fibroblasts by anticancer drugs or ionizing radiation (IR) (Tanaka et al., 1994) and for DNA damage-driven apoptosis in mitogen-activated T lymphocytes (Tamura et al., 1994). Induction of IRF-1 mRNA and protein levels, requires ATM (Pamment et al., 2002). However, *Atm*^−/−^ cells can still trigger the induction of IRF-1 in response to viral mimetics. Evidently, DDR and pro-inflammatory nuclear factors provide an ever-expanding functional network linking the cellular machineries that regulate the innate immune response and that sense and respond to DNA damage.

## Linking DDR and cell surface ligands

Most cells do not express cell surface ligands abundantly (Cerboni et al., 2014); however, such ligands are known to be up-regulated in cells exposed to stress, as well as in cancer cells (Smyth et al., 2005). Upon viral infection or malignant transformation, cells express MHC class I-like cell surface ligands e.g., MICA, MICB, ULBP1-6 of the activating immune receptor NKG2D. In humans, all NK cells and γδ T, αβ CD8 T cells, and NKT cells express NKG2D (López-Larrea et al., 2008) allowing them to detect and selectively remove damaged, unhealthy cells (Champsaur and Lanier, 2010). A direct link between NKG2D cell surface ligands and the DDR was shown in cells exposed to genotoxins. Most—if not all—of the NKG2D ligands tested, including *Raet1, Mult1*, and *H60a* genes in mice or MICA and ULBP genes in humans showed increased mRNA levels (Gasser et al., 2005; Gasser and Raulet, 2006). Although, it remains unknown how NKG2D ligands are induced upon DNA damage, this likely takes place at the posttranscriptional level (Himmelreich et al., 2011) and requires ATM and/or ATR along with downstream kinases, such as the checkpoint kinase (CHK) 1 and CHK2 (Gasser et al., 2005). In line, siRNA-mediated knockdown of ATM leads to the reduction of NKG2D ligand expression in cancer cells (Gasser et al., 2005) suggesting that ligand expression in tumors (that often bear chromosomal abnormalities) is driven by intrinsic genome instability rather than cellular transformation. Similar to NKG2D, DNAM-1 (DNAX Accessory Molecule-1), a 65 kDa transmembrane glycoprotein, is expressed in many cell types, including NK cells and some T cells (Shibuya et al., 1996). DNAM-1 promotes cellular adhesion to DNA damage-treated cells expressing DNAM-1 ligands (Soriani et al., 2009), such as CD155 and CD112, two adhesion molecules belonging to the Ig-like superfamily (Bottino et al., 2003). Other cell types such as fibroblasts, endothelial cells or lymphocytes and macrophages express cell surface glycoproteins such as the intercellular adhesion molecules (ICAMs). ICAM-1 is a transmembrane glycoprotein that serves as a ligand for lymphocyte function-associated antigen-1 (LFA-1) and macrophage antigen-1 (Mac-1), two receptors found on leukocytes that promote their adhesion to inflamed vascular endothelium and transendothelial migration (Yang et al., 2011). Whereas, ICAM-1 expression is suppressed upon UV irradiation-induced DNA damage (Ahrens et al., 1997), it is induced in response to IR in a p53-dependnent manner (Gaugler et al., 1997). Interestingly, P53 directly activates the expression of ICAM-1 in senescent cells in an NF-kB-independent manner (Gorgoulis et al., 2003). Macrophage, mast cells and dendritic cells also express a distinct type of pattern recognition receptors called the TLRs (Toll-Like Receptors) (Blasius and Beutler, 2010). TLRs recognize a spectrum of pathogen ligands, collectively referred to as PAMPs (Pathogen-Associated Molecular Patterns), most TLR promoters are targeted by P53 and can also be modulated by DNA damage with variation amongst individuals (Menendez et al., 2011).

## Linking DDR with cytokines

Cells carrying hallmarks of persistent DSBs may trigger the secretion of interleukins, such as interleukin-6 (IL-6) and interleukin-8 (IL-8) (Rodier et al., 2009). Release of IL-6 and IL-8 requires DDR i.e., ATM, NBS1, and CHK2; instead, the cell cycle inhibitors p53 or pRB are dispensable for the response (Rodier et al., 2009). NEMO and Receptor Interacting Protein (RIP) 1 kinase operate upstream of Il-6 and IL-8 secretion; ATM recruits NEMO and RIP1 through autocrine Tumor Necrosis Factor (TNF)-a signaling to trigger cytokine secretion and caspase activation (Biton and Ashkenazi, 2011). Upon exposure to UV irradiation, keratinocytes form large cytoplasmic complexes, called “inflammasomes” to trigger the maturation, activation and secretion of pro-inflammatory cytokines (Faustin and Reed, 2008; Schroder and Tschopp, 2010). At times, the presence of dsDNA derived from e.g., pathogen-damaged cells that are otherwise not exposed to any exogenous genotoxins may activate the stimulator of IFN genes (STING) and IRF3 (Kondo et al., 2013). In this case, the meiotic recombination 11 homolog A (MRE11) serves as the cytosolic sensor for the exogenous dsDNA. Once active, cytokines may instigate more DNA damage through the propagation and persistent maintenance of (chronic) inflammation (Jaiswal et al., 2000; Bartsch and Nair, 2006). Eventually, the inherent propensity of certain cells to secrete pro-inflammatory signals upon stress (Tchkonia et al., 2010) could establish self-perpetuating pro-inflammatory cycles leading to DNA damage and age-related diseases (Karakasilioti et al., 2013; Pateras et al., 2015), including cancer (Meira et al., 2008).

## DNA damage-driven inflammation and disease

Until recently, there would have been few examples to link DNA damage and inflammation to health and disease. However, recent findings allow us to consider several instances where innate immune responses driven by intrinsic genome instability or chronic exposure to exogenous genotoxins is causal to age-related degeneration, metabolic abnormalities and cancer (Coussens and Werb, 2002). Indeed, chronic inflammation is thought to generate an excess of reactive oxygen and nitrogen species (ROS, RNS) triggering DNA damage and malignancy (Wiseman and Halliwell, 1996; Kuper et al., 2000; Ohnishi et al., 2013). In support, chronic inflammation in the colon or the gastric cardia of mice is functionally linked to the formation of DNA lesions and the induction of the DDR, as well as with cancer induction (Meira et al., 2008; Lin et al., 2015).

Cellular senescence is a term used to describe cells that cease to divide in culture and has been one of the first paradigms to link DNA damage and immunity to disease (Campisi and d'Adda di Fagagna, 2007). Cellular senescence is often fueled by nuclear DNA damage followed by chronic DDR activation; telomere shortening, mitogenic oncogenes, or intrinsic DNA damage can lead to different types of senescence limiting the replicative lifespan of cells (Campisi and d'Adda di Fagagna, 2007). Persistent DNA damage and DDR signaling triggers senescent cells to secrete immunomodulatory proteins, a phenomenon known as the senescence-associated secretory phenotype (SASP) (Campisi and d'Adda di Fagagna, 2007; Fumagalli and d'Adda di Fagagna, 2009). SASP factors range from inflammatory and immune-modulatory cytokines to chemokines as well as growth factors, shed cell surface molecules, survival factors and extracellular matrix remodeling enzymes (Coppé et al., 2008; Ohanna et al., 2011; Acosta et al., 2013; Malaquin et al., 2013). Together, they impinge on cell-fate decisions in neighboring cells or the tissue microenvironment. For example, certain SASP factors, such as the CXCR2-binding chemokines reinforce growth arrest (Acosta et al., 2008) whereas other promote tumor clearance e.g., Csf1, Mcp1, Cxcl1, IL-15 (Xue et al., 2007), or growth e.g., IL-6 and IL-8 (Acosta and Gil, 2009). As DNA damage accumulates with age, persistent DDR-mediated release of SASP factors could be associated with degenerative changes that manifest with old age; in support, several SASP factors are considered amongst the most reliable biomarkers for age-related diseases (Fumagalli and d'Adda di Fagagna, 2009). In line, older individuals often show an increase in systemic inflammation (often termed “inflammaging”) as evidenced by the elevated levels of pro-inflammatory cytokines e.g., IL-6, clotting factors and acute phase reactants (Ferrucci et al., 1999; Cohen et al., 2003; Cavanagh et al., 2012; Shaw et al., 2013). Nevertheless, any direct evidence linking DNA damage to chronic inflammation stems from recent findings in progeroid (accelerated aging) syndromes and accompanying mouse models that carry inborn DNA repair defects. Patients with Werner syndrome (WS, associated with mutations in the RecQ DNA helicase) manifest with features of systemic chronic inflammation (Davis and Kipling, 2006), including the high serum levels of highly sensitive C-reactive protein (hs-CRP), an acute-phase protein of hepatic origin whose levels are increasing following interleukin-6 (Goto et al., 2012). Transcriptome analysis in cells lacking a functional CSB protein revealed an NF-κB-dependent pro-inflammatory response. The latter is thought to be responsible for the extraordinary neurodegenerative and wasting symptoms of this and other NER progeroid disorders (Newman et al., 2006; de Waard et al., 2010; Goss et al., 2011; Jaarsma et al., 2011; de Graaf et al., 2013; Barnhoorn et al., 2014). In other instances, DNA damage-driven inflammation may trigger tissue-specific degenerative changes leading to systemic metabolic abnormalities. Using animal models of the XFE human progeroid syndrome (Niedernhofer et al., 2006) that carry a DNA repair defect only in the adipose tissue (*aP2-Ercc1*^*F*/−^ mice), we recently showed that persistent DDR triggers a chronic auto-inflammatory response leading to severe fat depletion in mice (Karakasilioti et al., 2013). AP2-*Ercc1*^*F*/−^ fat depots showed hallmarks of persistent DDR together with the marked up-regulation of pro-inflammatory factors, the infiltration of activated macrophages as well as the release of DAMPs known to initiate and perpetuate immune responses (Karakasilioti et al., 2013). Further studies in aP2-*Ercc1*^*F*/−^ fat depots *in vivo* and in adipocytes *ex vivo* showed that persistent DNA damage signaling triggers the induction of IL-6, IL-8, and TNFα by promoting transcriptionally active histone marks and the dissociation of nuclear receptor co-repressor complexes from promoters; the response required ATM and it was instigated in a DNA lesion- and cell type-specific manner. In support of these findings, NF-κB is stochastically activated in tissues of naturally-aged and *Ercc1*^−/Δ^ mice (unlike *Ercc1*^−/−^ mice, the *Ercc1*^−/Δ^ animals maintain about 10% of the wild-type ERCC1 protein levels and develop progressive, degenerative changes that markedly resemble those seen in natural aging (Tilstra et al., 2012). Importantly, genetic depletion of the p65 subunit of NF-κB or pharmacologic inhibition of NF-κB delayed age-related symptoms in *Ercc1*^−/Δ^ mice. Moreover, inhibition of IKK/NF-κB activity reduced cellular senescence and oxidative damage in DNA and proteins (Tilstra et al., 2012). In other instances, the accumulation of prelamin A isoforms at the nuclear lamina triggers an ATM- and NEMO-dependent signaling pathway that leads to NF-κB activation and high levels of secreted pro-inflammatory cytokines in *Zmpste24*^−/−^ and *Lmna*^*G*609*G*/*G*609*G*^ progeroid animals. As in *Ercc1*^−/Δ^ animals, genetic and pharmacological inhibition of NF-κB signaling can ameliorate the age-associated features and extend the lifespan of these animal models (Osorio et al., 2012). Finally, *Atm*^−/−^ animals present with infiltration of neutrophils and lymphocytes in the lungs and increased mRNA levels of pro-inflammatory e.g., IL-6, TNF cytokines (Eickmeier et al., 2014).

ssDNA intermediates generated during e.g., transcription or DNA replication may also activate DDR and trigger a pro-inflammatory response (Abe et al., 2013). At any given time, proliferating cells may contain 1–2% of genomic DNA in single-stranded form (Bjursell et al., 1979). The relevance of ssDNA intermediates in humans is highlighted by the Aicardi–Goutières syndrome patients and mice that carry inborn defects in TREX1 (Three prime repair exonuclease 1); TREX1 degrades ssDNA polynucleotide species derived from the processing of aberrant DNA replication intermediates to prevent persistent DDR activation (Yang et al., 2007). The Aicardi–Goutières syndrome patients present with an auto-inflammatory phenotype leading to immune-mediated neurodevelopmental abnormalities (Chahwan and Chahwan, 2012) or cardiomyopathy and circulatory failure respectively (Coscoy and Raulet, 2007; Yang et al., 2007). Eventually, a universal theme arises from these recent findings; it is neither DNA damage nor senescence or cancer *per se* but persistent DDR that triggers the repertoire of innate immune responses (Fumagalli and d'Adda di Fagagna, 2009). Thus, any events that could potentially activate DDR could trigger the activation of innate immune responses in the absence of DNA damage; similarly suppressing DDR signaling in the presence of tolerable DNA damage levels could alleviate some of the pathological features associated with DNA damage-driven inflammation.

## Early benefits and late adverse consequences

**Figure 2:**
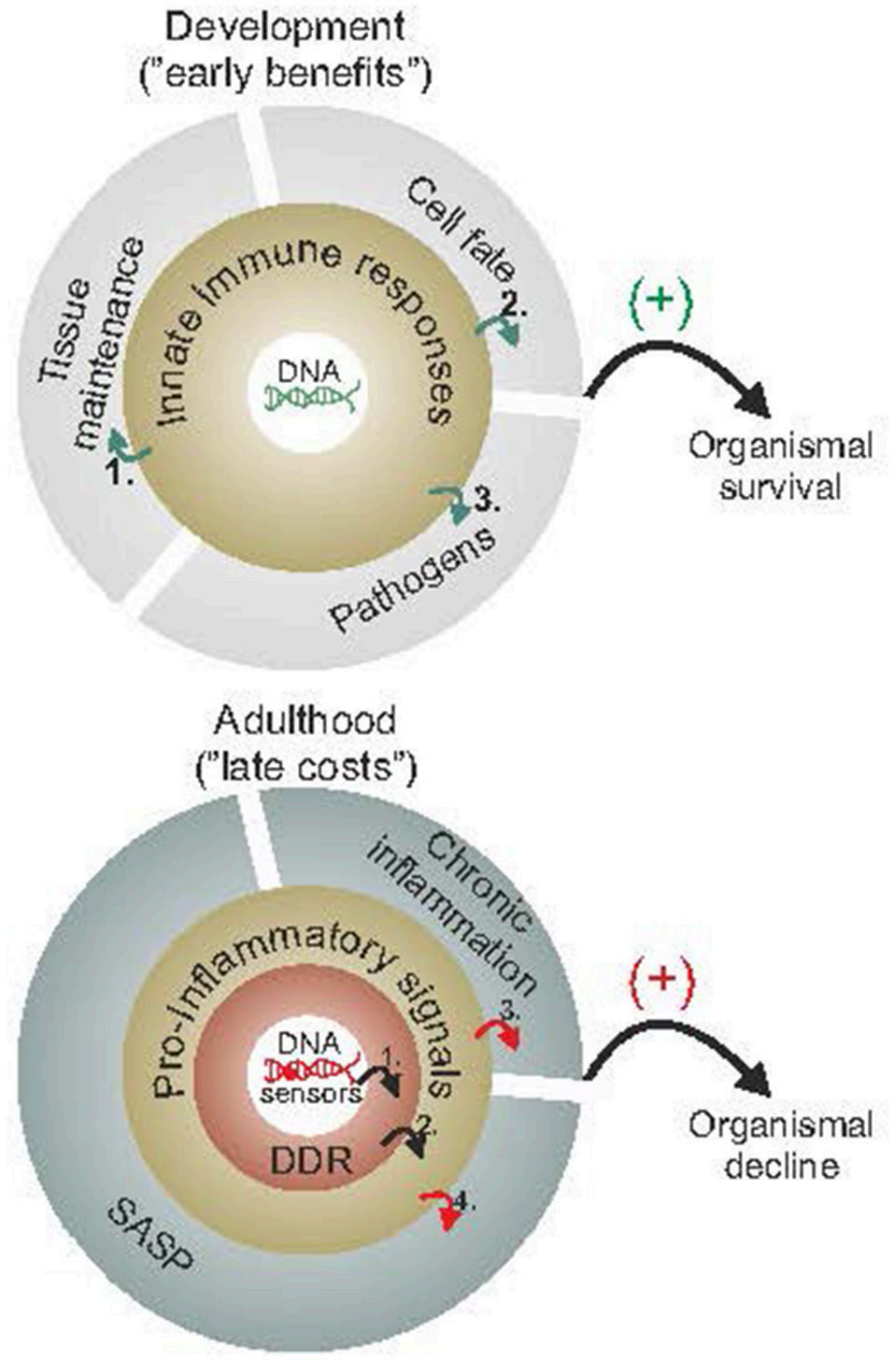
**Early benefits and late adverse consequences of innate immune responses**. Innate immune responses have been selected for by having their early benefits outweigh their late costs during evolution. As cells proliferate, grow or differentiate during the course of normal development (upper panel), they rely on maintenance and defense mechanisms to efficiently detect or remove damaged cells and promote wound healing **(1)**, fine tune cell-fate decisions, such as preventing cancer **(2)** or else defend against pathogens **(3)**. During adulthood and once reproductive maturity has been reached (lower panel), however, the competitive advantage to signal the presence of damaged macromolecules (as in youth) gradually deteriorates. The slow but steady buildup of DNA damage **(1)** is expected to trigger DDR and DDR-mediated pro-inflammatory stimuli **(2)** leading into a vicious cycle of persistent DDR, chronic inflammation **(3)** and SASP **(4)** with advancing age. The figure is a summarization/scheme of the manuscript.

DNA damage-driven inflammation can be both beneficial and detrimental for organismal survival (Figure 2). To understand this controversy, it may be helpful to consider that such responses have been selected for by having their early benefits outweigh their late costs during evolution. Early in life, priorities in mammals are shifted toward development, growth, and reproductive fitness. As cells divide, gain volume or differentiate, tissues rely on maintenance and defense mechanisms to efficiently detect and remove damaged cells. In doing so, specific cell types may activate immune responses to fine tune cell-fate decisions at the organismal level; for instance, DNA damage in germ cells induces an innate immune response in worms that promotes endurance of somatic tissues to allow delay of progeny production when germ cells are hit by DNA damage (Ermolaeva et al., 2013). Once reproductive maturity has been reached, the competitive advantage to signal the presence of damaged cells (in youth) is gradually deteriorating. Despite the efficiency of DNA repair mechanisms, some DNA damage is left unrepaired leading to the gradual accumulation of DNA lesions in cells. In turn, the slow but steady buildup of damaged cells within tissues is expected to intensify DDR responses over time. Likewise, the DDR-mediated pro-inflammatory signals may further alarm the neighboring cells and tissues for the presence of cells with compromised genome integrity. The latter triggers a vicious cycle of persistent DDR and pro-inflammatory signals leading to chronic inflammation, tissue malfunction and degeneration with old age; in DNA repair-deficient patients, the rapid accumulation of DNA damage (in view of the DNA repair defect) would trigger the untimely activation of DDR signaling leading to the early manifestation of age-related pathology that is associated with chronic inflammation.

Future strategies aimed at identifying new players or delineate key pathways may shed light on the biochemical crosstalk DNA repair and immune factors allowing us to gain insights onto how both systems contribute to disease origin and progression at old age. In this regard, the use of e.g., tissue-specific or tagged knockin animals and high-throughput proteomics and genomics approaches will likely prove valuable toward the development of rationalized interventions (Tilstra et al., 2012; Karakasilioti et al., 2013).

## Author contributions

AI and EG researched the literature, prepared a schematic first draft, and Figure 1. AI, EG and GG wrote the manuscript.

### Conflict of interest statement

The authors declare that the research was conducted in the absence of any commercial or financial relationships that could be construed as a potential conflict of interest.
